# Discounting the duration of bolus exposure in impedance testing underestimates acid reflux

**DOI:** 10.1186/s12876-016-0471-y

**Published:** 2016-06-08

**Authors:** Namasivayam Vikneswaran, Joseph A Murray

**Affiliations:** Department of Gastroenterology and Hepatology, Singapore General Hospital, Singapore, Singapore; Division of Gastroenterology and Hepatology, Department of Immunology, Mayo Clinic, 200 First Street SW, Rochester, MN 55905 USA

**Keywords:** Reflux, Impedance

## Abstract

**Background:**

Combined impedance-pH testing (MII) allows for detection of reflux episodes regardless of pH. However impedance-based diagnosis of reflux may not routinely account for duration of the reflux episode. We hypothesize that impedance testing may be less sensitive than pH-testing in detecting acid reflux off therapy as a result of discounting duration of exposure.

**Methods:**

Baseline characteristics and reflux parameters of MII studies performed off-anti-secretory medications were analyzed. Studies on acid suppressive medication and those with recording times less than 20 h or low baseline impedance were excluded.

**Results:**

A total of 73 consecutive MII studies were analyzed of which 31 MII studies had elevated acid exposure while 16 were abnormal by impedance criteria. MII testing off-therapy was more likely to be abnormal by pH criteria (percent time pH < 4) than impedance criteria (total reflux):[42 vs 22 % (*p* =0.02)]. Acid exposure (percent time pH < 4) identified more studies as abnormal than MII-detected acid reflux episodes [42 vs 34 % (*p* < 0.01)]. Mean acid clearance time (pH-detected) was significantly longer than median bolus clearance time (impedance-detected) in the total [98.7 s vs 12.6 s (*p* < 0.01)], upright [58.6 s vs 13.1 s (*p* < 0.01)], and recumbent positions [136.7 s vs 14.2 s (*p* < 0.01)] with the greatest difference seen in the recumbent position. The mean ratio of mean acid clearance time (pH-detected) and the median bolus clearance time (impedance-detected) was significantly higher in the recumbent position compared to the upright position [11. vs 5.3 (*p* = 0.01)].

**Conclusion:**

Ambulatory impedance testing underestimates acid reflux compared to esophageal acid exposure by discounting the prolonged period of mucosal contact with each acid reflux episode, particularly in the recumbent position.

## Background

Current treatment paradigms for GERD are based on the objective demonstration of pathologic acid exposure by reflux testing or endoscopic evidence of mucosal disease [1]. Conventional ambulatory esophageal pH monitoring defines reflux as an abrupt drop in distal esophageal pH below 4 with pathological acid exposure defined by a composite of 6 variables of which acid exposure time (percentage time with pH <4) is the single most useful and reproducible index that correlates with disease severity and treatment response [2–4]. Combined impedance-pH testing (MII) offers a potentially more precise means of measuring reflux by allowing differentiation of reflux from swallow-induced pH changes that may result from consumption of foods and liquids with pH < 4 as well as identifying non-acid reflux [5, 6] .

However pathological reflux in MII studies is defined by the number of impedance-detected reflux events and their temporal association with symptoms and may not routinely account for the duration of the reflux episode [7, 8]. Furthermore software analysis can be set to only analyze reflux events that have detected by impedance, thereby ignoring those events that have been deemed to be reflux by pH characteristics alone.

The duration of esophageal acid exposure measured by pH has been previously demonstrated to be longer than bolus duration determined by impedance [7]. We hypothesize that impedance testing may be less sensitive than pH testing in detecting acid reflux as it discounts the duration of bolus exposure and that this effect may be particularly significant in the supine position and under-diagnose pathological reflux in those not on acid suppressing medications.

## Methods

MII studies (Sandhill Scientific, Highlands Ranch, CO, USA) that were performed off-anti-secretory medications at Mayo Clinic, Rochester from 1 Jan 09 to 31 Mar 10 were retrospectively analyzed. MII studies were performed for the definitive diagnosis of pathological reflux in patients with refractory reflux symptoms, extra-esophageal reflux symptoms and for assessment prior to fundoplication. Ambulatory 24-h testing was performed after an overnight fast. The MII-pH catheter consists of a 2.1-mm polyurethane catheter incorporating 6 impedance segments and 1 pH-measuring electrode at 5 cm above the lower esophageal sphincter. Proton pump inhibitors and histamine-2 receptor antagonists were discontinued for 7 and 3 days prior to the test respectively. Patients undergoing testing are encouraged to maintain normal activities and sleep schedule. Post-test calibration of antimony pH probes is routinely performed to correct for pH drift [9]. Studies performed on acid suppressive medication, those with recording times less than 20 h and those with low baseline impedance reducing the sensitivity for detecting reflux events were excluded from analysis.

At the end of the 24 h recording period, data was transferred and analyzed manually using dedicated software (Bioview Analysis; Sandhill Scientific). All tracings were manually reviewed by the investigator [10]. Reflux events based on retrograde bolus events were initially identified by the analysis software and false positive events were deleted. Baseline characteristics as well as reflux testing parameters were analyzed including the following parameters - (1) the percentage of total time that the pH was < 4, (2) the percentage of upright time that the pH was <4, (3) the percentage of supine time that the pH was < 4, (4) the number of reflux events, (5) the number of reflux events longer than 5 min, and (6) the longest reflux event, (7) total number of impedance-detected reflux episodes (8) impedance-detected reflux episodes in the recumbent position (9) impedance-detected reflux episodes in the supine position. pH indices were compared with the corresponding impedance indices in the total, upright and recumbent positions. In particular, the reflux clearance characteristics were analyzed by comparing the mean acid clearance time (pH-detected) with the median bolus clearance time (impedance-detected) in the total, upright and recumbent position. The ratio of the mean acid clearance time (pH-detected) and the median bolus clearance time (impedance-detected) was analyzed in the upright and recumbent position to compare the magnitude of the discrepancy between pH and impedance in the upright and recumbent positions.

Distal esophageal acid exposure can be quantified using the percentage time pH < 4 or DeMeester score. MII studies were categorized as abnormal by acid exposure criteria if the percentage of total time that the pH was < 4 was 4.2 % or more [11]. MII studies were categorized as abnormal by impedance criteria if they had elevated number of impedance-detected reflux episodes (73 or more) [7]. The proportion of MII that were positive by impedance criteria was compared to the proportion of MII that were positive by acid exposure. In addition, the MII studies were separately categorized by acid exposure criteria on the basis of an elevated DeMeester score (14.7 or more) and the proportion of abnormal studies were compared with the proportion of abnormal MII by impedance criteria [11].

Baseline continuous data was compared using the 2-sample *t* test or the Wilcoxon rank-sum test depending on the data normality. Baseline categoric data was compared using the chi-squared test. Statistical analysis was performed using SPSS software (Statistical Package for the Social Sciences, Chicago, Illnois, USA). Statistical significance was defined as *p* value <0.05. This study was approved by Mayo Clinic Institutional Review Board. The waiver of the requirement to obtain informed consent was approved in accordance with 45 CFR 46.116.

## Results

A total of 73 consecutive MII studies performed off anti-secretory medication were analyzed. Of these, 50 were performed in females and the median age was 50 years (range 25–78). Baseline MII results are shown (Table 1). Thirty one MII studies had elevated acid exposure while 16 were abnormal by impedance criteria.

**Table 1:** Baseline MII results

	Parameters	Median	Range (Min, Max)
Duration of analysis	Duration of analysis total (hours:min)	21:22	(20:01, 24:22)
	Duration of analysis upright (hours:min)	12:17	(2:35, 18:25)
	Duration of analysis recumbent (hours:min)	8:59	(2:50, 20:47)
	Median gastric pH	2.2	(1.6,3.7)^a^
Esophageal acid exposure	Percentage time total ph < 4 (%)	1.6	(0,17.3)
	Percentage time upright ph < 4 (%)	1.9	(0,24.4)
	Percentage time recumbent ph < 4 (%)	0.8	(0,23.3)
	Episodes Over 5 min	0.0	(0,11.1)
	Longest Episode/min	4.8	(1,96.5)
	Total Episodes	27.2	(1.1, 122.7)
	Demeester score	5.8	(0.8,59.1)
Impedance	Baseline impedance/Ω	1614	(588,5420)
	no. of impedance events (total)	41	(6,162)
	no. of impedance events (upright)	37	(2,149)
	no. of impedance events (recumbent)	5	(0,45)
	longest impedance detected episode/min	3.4	(0.2,161)
	Acid reflux	21	(0,88)
	Non-acid reflux	17	(1,159)
	Bolus exposure (%)	1.3	(0.1, 21.2)

^a^Gastric pH data missing in 27 studies

Fifteen and thirty one studies had elevated acid exposure in the upright and recumbent position respectively. Fifteen and thirty one studies had increased impedance-detected reflux events in the upright and recumbent position respectively.

MII testing off-therapy was more likely to be abnormal by pH criteria (percent time pH < 4) than impedance criteria (total reflux):42 vs 22 % (*p* =0.02). Acid exposure (percent time pH < 4) identified more abnormal studies than MII detected reflux episodes: 42 vs 34 % (*p* <0.01).

Testing off therapy was more likely to be abnormal by DeMeester score than impedance criteria (total reflux): 41.1 vs 21.9 % (*p* = 0.01). The median longest episode detected by pH was significantly longer than the longest episode detected by impedance: 4.8 (0.1, 96.5) vs 3.4 (0.2, 161.4) minutes (*p* <0.01). The median total episodes of reflux detected by pH was significantly fewer than impedance-detected reflux episodes: 27.2 (1.1, 122.7) vs 41 (6,162) *p* < 0.01.

We further analyzed the reflux clearance characteristics by comparing pH versus impedance (Table 2). The mean acid clearance time (pH-detected) was significantly longer than the median bolus clearance time (impedance-detected) in the total, upright and recumbent positions: 98.7 s vs 12.6 s (*p* < 0.01), 58.6 s vs 13.1 s (*p* < 0.01), 136.7 s vs 14.2 s (*p* < 0.01) with the greatest difference seen in the recumbent position. The mean ratio of mean acid clearance time (pH-detected) and the median bolus clearance time (impedance-detected) was significantly higher in the recumbent position compared to the upright position: 11. vs 5.3 (*p* = 0.01). This indicates that the difference between pH detected acid clearance and impedance-detected bolus clearance is greater in the recumbent position compared to the upright position.

**Table 2:** Temporal characteristics of acid clearance versus impedance detected bolus clearance

	Parameters	Median	Range (Min, Max)
Esophageal acid exposure	Mean Acid Clearance time - total (sec)	66	(7,1257)
	Mean Acid Clearance time - upright (sec)	48	(0,179)
	Mean Acid Clearance time - recumbent (sec)	44	(0,1257)
Impedance	Median Bolus Clearance Time-total/sec	12	(3,31)
	Median Bolus Clearance Time-upright/sec	12	(5,37)
	Median Bolus Clearance Time- recumbent/sec	10	(0,96)

## Discussion

Standard pH- only ambulatory esophageal monitoring has been largely replaced with combined impedance-pH monitoring on the premise that impedance provides a diagnostic gain by detecting non-acid reflux and improving symptom reflux correlation during testing on therapy [12–14]. Impedance monitoring has expanded on pH-metry with detection of reflux of liquid irrespective of acidity and gas which has enabled detection of reflux on anti-secretory therapy as well as characterization of non-reflux conditions such as rumination and supragastric belching [15, 16].

However, impedance-based categorization of reflux based on normative data has not been validated by outcome-based studies and recent outcome-based studies suggest impedance parameters may not be as predictive as conventional acid exposure in predicting treatment response [17, 18]. The true relevance of negative impedance studies performed on antisecretory therapy without prior confirmation of reflux is also unclear.

Our study demonstrates that in testing off therapy, impedance underestimates reflux compared to esophageal acid exposure resulting in a decreased proportion of studies positive for pathological reflux. Both acid exposure (pH <4) as well as DeMeester score were more likely to result in a positive test than impedance detected reflux episodes. This is despite the increased detection of reflux episodes by impedance compared to conventional pH criteria.

This may be due to the failure of impedance to account for the prolonged period of mucosal contact with each acid reflux episode particularly in the recumbent position. The mean acid clearance time (pH-detected) was significantly longer than the median bolus clearance time (impedance-detected) in all positions. Furthermore, the median longest episode was significantly longer with pH testing compared to impedance. These findings indicate that impedance underestimates the duration of bolus exposure to esophageal mucosa compared to pH. These differences are more marked in the recumbent position since the ratio of pH detected mean acid clearance time and impedance detected median bolus clearance time was significantly higher in the recumbent position compared to the upright position.

These findings are consistent with the two-step acid clearing mechanism. Esophageal acid clearance is initiated by peristalsis and completed following neutralization by swallowed alkaline saliva. Impedance returns to baseline values with clearance of the majority of the acid bolus by peristalsis but pH detects the small amount of residual acid that persists until neutralization by alkaline saliva [19]. This difference is more marked in the recumbent period where the much longer acid reflux events are not adequately reflected by the impedance measures. This is likely due to the lack of buffering by alkaline saliva as the production of saliva is reduced at night. Decreased swallowing frequency, salivary production and decreased delivery to the distal esophagus during sleep retards neutralization of esophageal pH after acid reflux [20]. So, the pH does not normalize as quickly due to the decreased saliva even though bolus clearance by impedance is almost equal in the upright and recumbent position as demonstrated in this study. Hence in studies performed off medication, the pH component appears to be more reliable particularly in the recumbent position. Studies with low baseline impedance that could confound analysis by lowering the sensitivity of impedance detection of reflux were excluded hence this could not account for the results.

Nocturnal acid reflux can induce sleep disturbances and decrease health-related quality of life [21, 22]. Furthermore nocturnal acid reflux has been associated with more severe esophageal mucosal damage, such as peptic stricture and adenocarcinoma and an increased risk of pulmonary complications [23, 24]. Under-diagnosis of reflux may have significant public health implications.

In the absence of acid suppression, clinicians need to pay more attention to pH parameters than impedance. Perhaps impedance-based diagnostic criteria need to be revised to accurately reflect duration of acid contact especially in the recumbent position. Current analysis software may calculate impedance-detected bolus exposure. Although normative data on impedance-derived bolus measures is available and could potentially be incorporated into impedance-based diagnosis of reflux, emerging studies suggest that impedance parameters may not be as predictive as conventional acid exposure in predicting treatment response [17, 18]. Future studies could compare pH- and impedance-detected bolus exposure to assess the impact of incorporating impedance-detected bolus exposure on the impedance-based diagnosis of reflux.

The findings may have implications for testing on therapy that merit further study. Patients with prolonged non-acid reflux events below the current impedance-based diagnostic thresholds may still have clinically significant reflux events that could account for symptoms even if they may not be as injurious to the mucosa.

There are shortcomings to this study. This is a retrospective review. Recumbency was also assumed to be a surrogate for sleep when saliva production in decreased though differences in reflux characteristics of recumbent-awake and recumbent-asleep periods are not routinely measured in clinical practice [25].

## Conclusion

In conclusion, impedance underestimates acid reflux compared to esophageal acid exposure with testing off therapy. This may be due to the failure of impedance to account for the prolonged period of mucosal contact with each acid reflux episode particularly in the recumbent position. Clinicians should continue to rely on standard pH parameters for assessment of reflux in those patients being studied off acid suppressive medications.

## Abbreviations

MII, Combined impedance-pH testing
